# Prediction by Graph Theoretic Measures of Structural Effects in Proteins Arising from Non-Synonymous Single Nucleotide Polymorphisms

**DOI:** 10.1371/journal.pcbi.1000135

**Published:** 2008-07-25

**Authors:** Tammy M. K. Cheng, Yu-En Lu, Michele Vendruscolo, Pietro Lio', Tom L. Blundell

**Affiliations:** 1 Department of Biochemistry, University of Cambridge, Cambridge, United Kingdom; 2 Computer Laboratory, University of Cambridge, Cambridge, United Kingdom; 3 Department of Chemistry, University of Cambridge, Cambridge, United Kingdom; National Cancer Institute, United States of America, and Tel Aviv University, Israel

## Abstract

Recent analyses of human genome sequences have given rise to impressive advances in identifying non-synonymous single nucleotide polymorphisms (nsSNPs). By contrast, the annotation of nsSNPs and their links to diseases are progressing at a much slower pace. Many of the current approaches to analysing disease-associated nsSNPs use primarily sequence and evolutionary information, while structural information is relatively less exploited. In order to explore the potential of such information, we developed a structure-based approach, *Bongo* (Bonds ON Graph), to predict structural effects of nsSNPs. *Bongo* considers protein structures as residue–residue interaction networks and applies graph theoretical measures to identify the residues that are critical for maintaining structural stability by assessing the consequences on the interaction network of single point mutations. Our results show that *Bongo* is able to identify mutations that cause both local and global structural effects, with a remarkably low false positive rate. Application of the *Bongo* method to the prediction of 506 disease-associated nsSNPs resulted in a performance (positive predictive value, PPV, 78.5%) similar to that of *PolyPhen* (PPV, 77.2%) and *PANTHER* (PPV, 72.2%). As the *Bongo* method is solely structure-based, our results indicate that the structural changes resulting from nsSNPs are closely associated to their pathological consequences.

## Author Summary

Non-synonymous single nucleotide polymorphisms (nsSNPs) are single base differences between individual genomes that lead to amino acid changes in protein sequences. They may influence an individual's susceptibility to disease or response to drugs through their impacts on a protein's structure and hence cause functional changes. In this paper, we present a new methodology to estimate the impact of nsSNPs on disease susceptibility. This is made possible by characterising the protein structure and the change of structural stability due to nsSNPs. We show that our computer program *Bongo*, which describes protein structures as interlinked amino acids, can identify conformational changes resulting from nsSNPs that are closely associated with pathological consequences. *Bongo* requires only structural information to analyze nsSNPs and thus is complementary to methods that use evolutionary information. *Bongo* helps us investigate the suggestion that most disease-causing mutations disturb structural features of proteins, thus affecting their stability. We anticipate that making *Bongo* available to the community will facilitate a better understanding of disease-associated nsSNPs and thus benefit personal medicine in the future.

## Introduction

The introduction of large-scale genome sequencing technologies has dramatically increased the number of single nucleotide polymorphisms (SNPs) in public databases. For example, the NCBI (National Center for Biotechnology Information) dbSNP database [1], which is a major repository of human SNPs, contained data about ten thousand unique human SNPs as of Build 106 in 2002. By October 2007, there were about six and half million validated unique human SNPs, as of Build 128. Although the progress of collecting SNP data has been impressive, the pace at which disease-related SNPs are annotated is much slower. So far, only a few thousand SNPs have been associated with a human genetic disorder in the OMIM (Online Mendelian Inheritance in Man) database [2]. Further efforts are thus required to identify disease-associated SNPs in order to understand their effects on human health.

Genetic variations, such as SNPs, are likely to contribute to susceptibility to complex diseases such as cancer [3]. Single nucleotide variations in the coding regions that lead to amino acid substitutions, the so-called non-synonymous SNPs (nsSNPs), may be associated with a modulation of protein function. For example, extensive studies on point mutations in P-glycoprotein have shown that amino acid variations regulate its substrate specificity and lead to a variation of drug disposition among individuals [4]. As a consequence, attention has been focused on the study of the relation between nsSNPs and disease as well as predicting their phenotypic effects. Some early approaches exploited position-specific evolutionary information contained in multiple sequence alignments [5],[6]. Others have used predictive features of sequence and structure [7],[8], or machine learning algorithms [9]–[11] to classify SNPs. In addition, there are approaches that annotate nsSNPs at a genomic scale, such as LS-SNP [12]. Previous analyses have shown that methods that apply only sequence information may suffer significant reductions in accuracy when fewer than ten homologous sequences are available for the target protein [8]. Sunyaev et al. [13] have shown that disease-causing mutations often affect intrinsic structural features of proteins, while in an important study Wang and Moult [14] have demonstrated that most disease-associated mutations appear to affect protein stability rather than interfere directly with protein interactions. Following these results, others have focused on comparing the structures of wild-type and mutant-type proteins [14],[15] or have estimated the change of protein stability by using environment-specific amino acid substitution matrices that are derived from the three-dimensional structures of homologous proteins [16].

For analyzing structural effects of nsSNPs, we have developed an approach, *Bongo* (Bonds ON Graph, http://www-cryst.bioc.cam.ac.uk/˜tammy/Bongo), which uses graph theoretic measures to annotate nsSNPs. Graph theory has found many applications in the study of protein structures during the past two decades. For example, Ahmed and Gohlke used graphs to identify rigid clusters for modelling macromolecular conformational changes [17]; Canutescu and colleagues have predicted side-chain conformations by partitioning graphs in which vertices represent residues [18]; Vendruscolo and colleagues applied small-world networks to identify key residues that are important for protein folding [19]; Jacobs, Thorpe and their colleagues used graphs to describe bond-bending networks between atoms, so identifying the rigid and flexible regions in the proteins [20],[21]; Kannan [22]; and Brinda and Vishveshwara [23] used the graph spectral method to identify side chain clusters that are important for protein folding and oligomerisation ; Sol and colleagues used graphs to identify key residues for allosteric communication and modular connection by the edge betweenness algorithm [24],[25]. *Bongo* uses graphs to represent residue-residue interaction networks within proteins and to assign key residues that are important for maintaining the networks. The novelty lies in the application of a graph theory concept, *vertex cover*, by which key residues are identified for analyzing structural effects of single point mutations.

Here we begin by describing the use of interaction graphs to represent protein structures. We then introduce the ‘key residues’ that *Bongo* uses to evaluate structural impacts of point mutations, and explain their roles in terms of stabilising protein structures. We further describe the algorithm of *Bongo*, where a graph concept *vertex cover* was adapted to identify key residues, and we calibrate *Bongo* over eight single point mutations that result in a range of different structural changes in the p53 core domain. We evaluate the false positive rate of *Bongo* for 113 mutations where wild-type and mutant-type crystal structures have been demonstrated to have negligible differences in backbone conformation. Eventually, we evaluate the performance of *Bongo* by testing its ability to distinguish disease- and non-disease-associated nsSNPs in protein structures in the PDB (Protein Data Bank) [26]. Based on the benchmark results, we also analyse the percentage of disease-associated nsSNPs that are likely to cause structural effects in proteins.

## Results/Discussion

### *Bongo* Considers the Long-Distance Structural Impact of a Point Mutation

A point mutation in a protein may often give rise only to a rearrangement of amino acid side chains near the mutation site, although sometimes a more substantial movement of polypeptide backbone locally or globally results. The former changes can be analysed by looking at the inter-residue interactions that a mutation creates or abolishes between its neighbouring residues. However the same approach may not be applicable to the latter, since simply paying attention to interactions immediately around a mutation site is not sufficient to predict structural effects on a larger scale.

**Figure 1:**
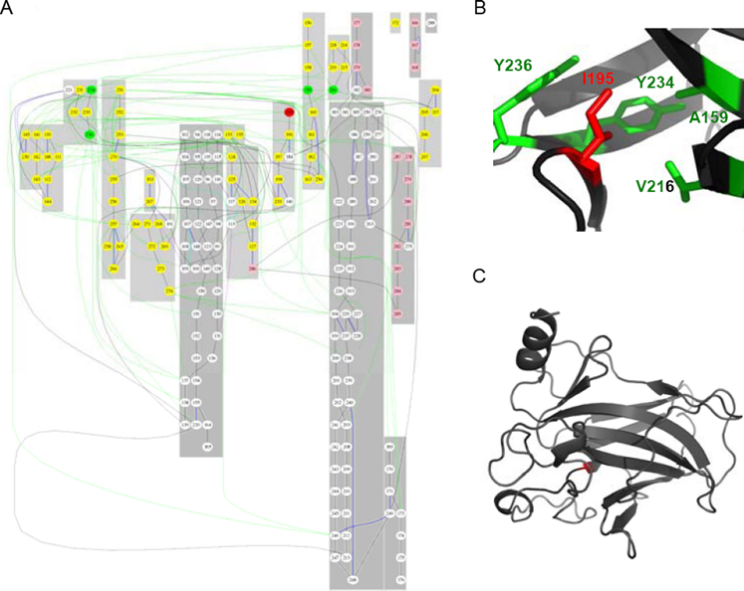
The graph model of *Bongo*. (A) A graph that represents the residue-residue interaction network in the p53 core domain. Each vertex in the graph represents a residue: the pink ones are in α-helices; the yellow are in β-strands and the white are in loops. The edges with different colors represent different interactions: blue for hydrogen bonds; cyan for π–π interactions; purple for π-cation interactions; green for hydrophobic interactions; black for backbones. The grey patches indicate segments of secondary structures, patches that are too close to each other can not be separated in the graph. (B) Residue I195 in p53 core domain has non-polar interactions with residues A159, V216, Y234, and Y236, and these local hydrophobic interactions are transformed into graph (A), where I195 is shown as a red vertex and A159, V216, Y234, and Y236 are shown as green vertices. (C) The overall structure of p53 core domain, where the location of I195 is shown in red.

In order to understand structural changes at a longer distance, we represent a protein as a residue-residue interaction graph, in which vertices represent residues and edges represent interactions between residues (Figure 1) (see more details in Methods). Of course, molecular dynamics calculations provide a powerful tool for identifying the impact of point mutations on the stability of the native states of proteins. However, these simulations are often time-consuming and require large computer power. Thus we have developed *Bongo* to provide an alternative approach by operating on interaction graphs, which are computationally more convenient. In our model, residue-residue interactions occur either through direct connection or through indirect links that involve intermediate residues. Such connectivity is based on ‘key residues’ that are important in maintaining the overall topology of the network, and thus the stability of the folded structure. These key residues eventually serve as reference points to evaluate whether a mutation can induce structural changes in a protein away from the mutation site.

### *Bongo* Measures the Structural Impacts by Comparing the Key Residues in the Interaction Graphs

*Bongo* measures the impact of a mutation according to its effects on key residues; it formulates the structural changes in a protein as changes of the key residues in a corresponding interaction graph. Here we adapt a variant of the *vertex cover*, defined in graph theory as a minimum set of vertices (residues) that are crucial to forming all the edges (interactions), to represent the key residues.

**Figure 2:**
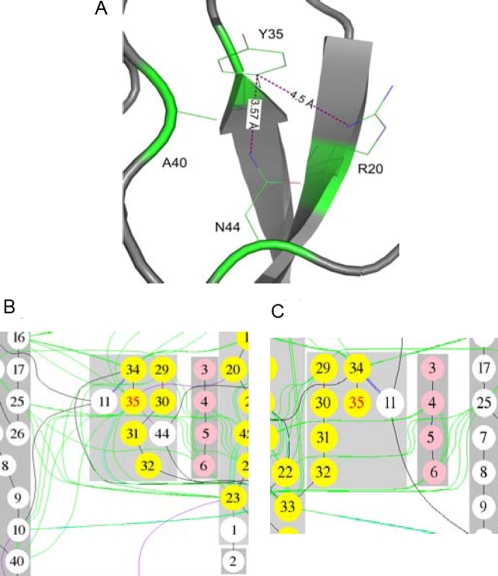
An example showing local structural changes between wild-type and mutant-type proteins. (A) Local environment around residue Y35 in protein 1BPI. (B) Wild-type local interaction graph around residue Y35. The interactions with residue R20, N44, and A40 are marked in the same colours as in (A). (C) Mutant-type local interaction graph around position 35.

In Figure 2, we illustrate the notion of key residues and introduce the use of the difference between the vertex cover of wild and mutant type interaction graphs as a measure of the effects of a mutation. The example here is residue Y35 of protein 1BPI, a key residue forming several relatively strong interactions including pi-cation interactions with residues R20 and N44 and a hydrophobic interaction with residue A40 (Figure 2A and 2B). The mutation Y35G removes this amino acid from the set of key residues in the graph (Figure 2C) as its original interactions with other secondary structure elements no longer exist. Hence, residue 35 is no longer a key residue in the mutant interaction network. Therefore, this mutation is considered structurally damaging by *Bongo*; we discuss the exact criteria under which a mutation is deemed damaging below.

### Rationale for Identifying Key Residues through the Vertex Cover

*Bongo* derives the interaction graph of a protein by considering each residue as a vertex and each residue-residue interaction, including hydrogen bonds, π–π, π–cation, and hydrophobic interactions, as an edge. The weight on each edge differs according to the total number of cross-secondary structure interactions as well as number of interactions with individual residues. The weighting scheme was calibrated against eight disease-associated mutations in the p53 core domain analysed by Fersht and co-workers [27],[28], as shown in Table 1. The optimised weighting of inter-secondary structure interactions is 0.8, 0.8, 0.8, 2.0 and 2.0 for H-bonds, π–π, π–cation, hydrophobic interaction, and hydrophobic core respectively. For internal interactions, H-bonds, π–π, π–cation interactions were given a weight of 0.6 and hydrophobic interaction a weight of 0.8. This distinction between inter and intra secondary structure interactions is used to reflect concerted movement of structural motifs within proteins. Thus, a single interaction loss among two densely interacting structures is less significant than one among two sparsely interacting ones.

**Table 1:** Prediction of nsSNPs in the core domain of p53 (PDB: 1TSR) by *Bongo*

Mutant categories	nsSNP	ΔΔ*G* a (kcal/mol)	Crystal structure	Prediction of *Bongo*	Prediction of *PolyPhen*
No structural effects	R273H	0.09 [28]	2BIM	Benign	Probably damaging
Weakly/locally destabilising	G245S	1.22 [34]	–b	Damaging	Probably damaging
	R249S	1.69 [34]	2BIO	Damaging	Probably damaging
	R248A	1–2 [28]	–	Damaging	Probably damaging
Highly destabilising/global unfolding	C242S	>2 [28]	–	Damaging	Probably damaging
	H168R	2.75 [34]	2BIN	Damaging	Probably damaging
	V143A	3.34 [34]	–	Damaging	Benign
	I195T	>2 [28]	–	Damaging	Probably damaging

a The free energy difference (destabilisation) compared to wild-type p53 core domain.

b “–” means no mutant crystal structure available.

**Figure 3:**
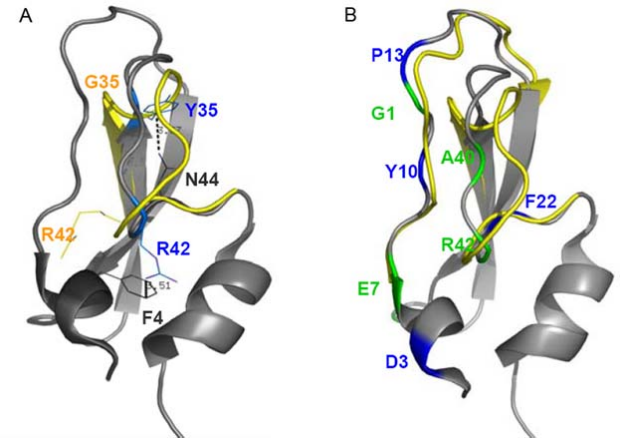
nsSNP Y35G in protein 1BPI. (A) Key residues (in blue) whose interactions are changed when the mutation Y35G is introduced into protein 1BPI. The key residue Y35 (upper) has a pi-cation interaction with residue N44 in the wild-type structure (shown in grey) and the interaction is abolished when the mutation happens (the mutant-type structure is shown in yellow). The key residue R42 (lower) has a π–cation interaction with residue F4 in the wild-type structure and the interaction is abolished when the mutation happens (the corresponding position of R42 in the mutant structure is shown with a yellow side chain). (B) Key residues that are specific in the modelled mutant-type structure are shown in blue, while those are specific in the crystal structure are shown in green. The wild-type structure of 1BPI is shown in grey, while the region that under conformational change due to the mutation Y35G is shown in yellow.

Based on the above weighting scheme, *Bongo* defines the key residues as the *minimum weighted vertex cover* (see the definition of vertex cover in Methods), which represents the minimum necessary residues to establish the interaction network. However, finding the minimum vertex cover is known to be NP-complete and hence efficient algorithms only exist for approximate solutions [29]. Therefore, we use a selection scheme which adopts an approximation algorithm based on the greedy principle to identify the key residues. The approximation algorithm is known to give vertex covers that cost no more than H(|V|), where |V| denotes the size of a vertex set, times than the optimum solution where H(n) is the *n*th harmonic number. Compared to other graph theoretic constructs such as dominating sets [29], the vertex cover gives an intuitive notion of vertex importance. In fact, we have used more advanced techniques such as spectral decomposition [29] to identify structural information that is related to protein stability change, ΔΔ*G*. However, the results were not better than those obtained by applying the vertex cover approach (data not shown). Indeed, we have observed in some cases (Figure 3) that the change of vertex cover after mutation correlates well with structural data. Therefore, we believe that the vertex cover can serve as a useful approach to estimating protein structural changes.

### The Structural Role of Key Residues

**Figure 4:**
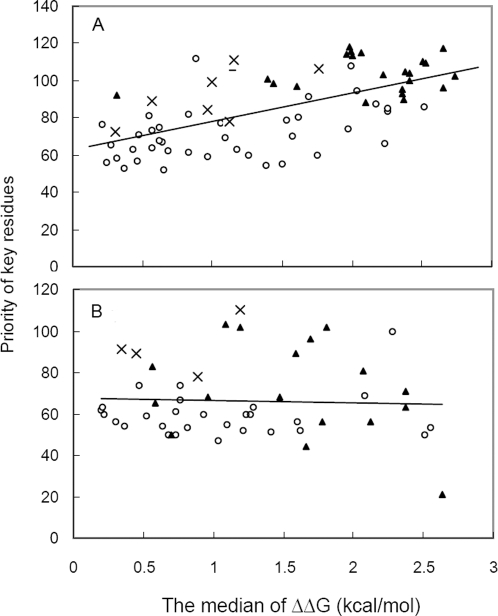
Correlation between the residue priority and the stability change ΔΔ*G* in the p53 core domain. (A) Correlation between the key residue priority and the stability change ΔΔ*G* of key residues. (B) Correlation between the assumptive priority and the stability change ΔΔ*G* of non-key residues. Open circle markers represent key residues in loops, triangle markers represent key residues in strands, and cross markers represent key residues in helix.

The key residues maintain the interaction networks in a protein, and each is assigned a priority value that measures its importance in determining the overall topology of the network (see Methods). When a point mutation is introduced into a protein, *Bongo* quantifies its structural effects according to the priorities of key residues affected. Thus we expect key residues, especially those with high priorities, to have important roles in stabilising folded protein structures. In order to check if the priority of key residues reflects their roles in forming structures, we calculated the correlation between the priority and the stability change (Each key residue was mutated to 19 other amino acids and the stability changes were calculated by I-mutant2.0 [30] (http://gpcr2.biocomp.unibo.it/˜emidio/I-Mutant2.0/I-Mutant2.0_Details.html), which has accuracy around 80% for predicting stability changes resulting from mutations when the three-dimensional protein structure is known. We consider only mutations that cause |ΔΔ*G*|<3kcal/mol since they affect the stability without totally abolishing the overall structure of the protein. The median number of |ΔΔ*G*|<3kcal/mol is used to calculate the correlation with the priority of key residues in order to avoid data skewness.), ΔΔ*G*, of key residues identified from the p53 core domain (PDB: 1TSR). When we considered the top half of the key residues ranked by their priorities, ΔΔ*G* relates to the priority of key residues with a Pearson correlation *r* = 0.61 and a significantly small *p*-value less than 0.001 (Figure 4A). This indicates that the correlation is statistically significant and also shows a good contrast to the low relation (*r* = −0.04) between assumptive priority (Since the non-key residues do not have priority values, they are assigned values according to those of the key residues that are nearest in the same secondary structures. If a non-key residue is flanked by two key residues, its assumed priority is the average of the priority values of its two neighbours.) and ΔΔ*G* of non-key residues (Figure 4B).

We noticed that the correlation is weaker (*r* = 0.36) when the lower half of key residues, ranked by their priorities, is included. This is likely due to uncertainties in the definitions of key residues that are ranked with lower priorities: Since *Bongo* stops selecting key residues only when no edges are left in a graph, the key residues that have lower priorities may not have structural meaning but are simply chosen in order to complete the selection process (covering all the edges/interactions in the graph). In an attempt to exclude the uncertain key residues, we analysed how far the correlation is valid by gradually including key residues that have priorities in the lower half, in order of decreasing priorities. There is an acceptable correlation *r* = 0.52 when we consider up to three fourths of overall key residues, which suggests that the bottom one quarter key residues are not reliable indicators of structural effects. Thus *Bongo* does not consider the bottom quarter key residues so that their uncertainty does not affect the prediction results.

The distribution of key residues according to their location in secondary structures (Figure 4A) shows that the key residues in β-strands tend to have larger ΔΔ*G*s and priority values compared to those in loops, whereas such differences are less clear for the case of non-key residues (Figure 4B). This suggests that, in general, protein stability should be more vulnerable to mutations in β-strands than those in loops, consistent with the observation that the β-strands in the p53 core domain are the major contributors to the core region of the protein. It also indicates that priority values and ΔΔG of key residues have consistent meanings in terms of protein structure.

### *Bongo* Evaluates the Structural Impact through the Resulting Changes in the Key Residues

Since the structures of the mutant proteins are not often available for nsSNPs, *Bong*o first uses *Andante* [31] to model the mutant-type protein structure by rearranging the side chain around the mutation site. The structural effects of a mutation are then analysed by comparing the wild-type and mutant-type key residues, denoted as *K*_wt_ and *K*_mt_, respectively. If a key residue in *K*_wt_ is not found in *K*_mt_, then it is considered to be affected by the mutation. Consequently the overall impact (*I*) of a mutation is calculated according to the key residues affected by the mutation, i.e.
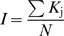
(1)where *I* is the total impact value, *K*_j_ is the priority of each key residue that is in *K*_wt_ but not in *K*_mt_. *N* is the total number of key residues in *K*_wt_, which normalise the size of proteins.

**Figure 5:**
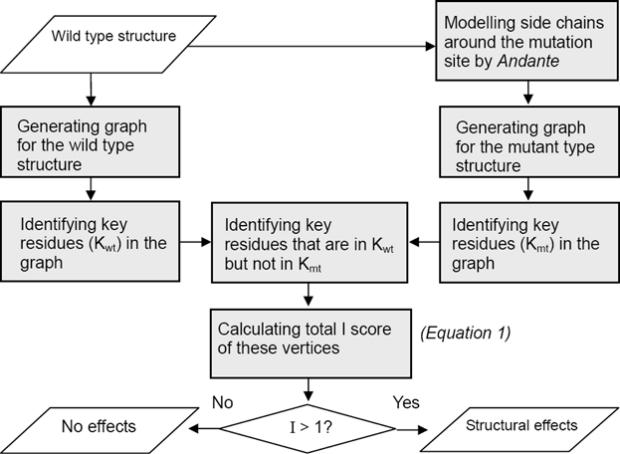
The flowchart of *Bongo*. Scheme showing how *Bongo* works (see text for details).

Thus each mutation is systematically quantified by its impact value I (an overview scheme of *Bongo* is shown in Figure 5). On deriving the impact value, *Bongo* considers mutations with *I*>1 to cause structural effects, which is the criterion calibrated over mutations in the p53 core domain.

In Figure 3, we give an example, the mutation Y35G in protein 1BPI, of how a mutation can have significant impact value. In addition to residue Y35, *Bongo* also predicts residues R42 to be affected by the mutation (Figure 3A). These two are at the ends of β strands and also in long loops linked to them. These regions undergo conformational changes when the mutation Y35G is introduced into the protein, where the biggest movement (4.2 Å) occurs between the wild-type and mutant-type Cα atom of residue G36 (The movement is measured when the wild-type (1BPI) and the mutant-type (8PTI) are superimposed by their Cα atoms.). Since the impact score calculated on the basis of these residues is greater than one, *Bongo* considers the mutation Y35G to cause structural effects in 1BPI, which corresponds to the experimental result.

In order to assess the errors due to the difference of a crystal structure of the mutant and a simulated one, we also compared the key residues of the two structures. It turns out that the differences of key residues between the modelled and the crystal structures are mostly located in the loop region, where structural changes occur when the mutation is introduced into the protein (Figure 3B). The overall distribution of the key residues that are specific for the modelled structure is similar to that of the key residues specific for the crystal structure. This suggests that the structural change at a longer distance can be captured in the interaction graphs by simply modelling a point mutation as rearrangement of side chains neighbouring to the mutation site.

### Calibration of *Bongo* by Mutations in the p53 Core Domain

In order to calibrate *Bongo*, we have used experimental data on the tumour suppressor p53 core domain, which is responsible for about 50% of mutations that lead to human cancers [32]. Owing to its importance, the wild-type and many mutant protein crystal structures have been determined. Several studies have been carried out for these point mutations within the domain, and thus make it a good calibration system for predicting structural effects of mutations. Furthermore, the structure of the p53 core domain is inherently unstable with a melting temperature of ∼42–44°C [33]. As a consequence, point mutations that cause either subtle structural changes or more dramatic effects are available for comparison.

**Figure 6:**
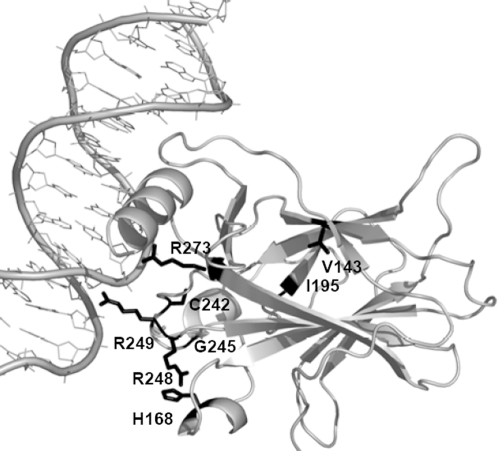
The eight nsSNPs that are listed in Table 1. Structure of p53 core domain is shown in grey at right; DNA is shown in grey at left; the nsSNPs are shown in black sticks.

For our study we identified eight nsSNPs (Figure 6) analysed experimentally by Fersht and co-workers [27],[28]. These mutations involve several different levels of structural change in the p53 core domain: (i) R273H has only a minor effect on the overall structure, with root mean square deviation (RMSD) ≤0.21Å in Cα positions between wild type and mutant type crystal structures; (ii) G245S, R249S, and R248A destabilise the p53 core domain by 1–2 kcal/mol and lead to local structural changes; (iii) C242S, H168R, V143A, and I195T destabilise the structure >2 kcal/mol and lead to global unfolding of the protein at body temperature. When the structure 1TSR in PDB was used as a calibration model, *Bongo* identified all mutations except R273H as causing structural effects in the p53 core domain (Table 1), which corresponds well with experimental data described in the literature.

For comparison we also used *PolyPhen* [5] to predict the effects of the same mutations. We consider *PolyPhen* as it uses multi-source data including three-dimensional structures, sequence alignments and SWISS-PROT annotations. Compared to other methods which either focus on protein structure or sequence information, it provides more comprehensive results. Of course, there are other methods that include even more information—for example, LS-SNP [12] also considers functional pathways, domain–domain interfaces, ligand–protein binding—but our purpose is to understand the usefulness of structural information by comparing it with a standard approach that mainly uses sequence and structural information. The results in Table 1 show that *PolyPhen* predicts all mutations except V143A to be probably damaging. *PolyPhen*'s success in predicting R273H to be damaging is probably a consequence of the fact that R273 is functionally important for binding DNA and thus conserved in sequence for reasons that are not evident from consideration of the structure alone, whereas *PolyPhen* predicts V143A to be benign, probably as a result of comparatively weaker emphasis on structural information.

### *Bongo* Has a Low False Positive Rate in Predicting Structural Effects

We further tested the application of *Bongo* to single point mutations that do not affect protein structure. Our benchmark set included 113 pairs of wild-type and mutant-type crystal structures in which each of them has RMSD in their backbone Cα atoms <0.4Å and the lower resolution of the two structures is ≤2.2Å (Dataset S1A). We chose these criteria in order to allow for experimental errors in the crystallographic solution of the structures of identical proteins, as suggested in the work of Hubbard and Blundell [35]. The benchmark result shows that *Bongo* predicts three of the single point mutations to cause structural effects, therefore yields a 2.7% false positive rate. Although this result may not be generalised to all the cases, it indeed encourages us to expect a low false positive prediction rate.

### Evaluation of *Bongo* over Disease- and Non-Disease-Associated nsSNPs

In the previous sections, we have shown that *Bongo* is able to predict structural effects of single point mutations with a low false positive rate. Here we further analyse the performance of *Bongo* in identifying disease-associated nsSNPs. Our test-set contains 506 disease-associated nsSNPs from the OMIM (Online Mendelian Inheritance in Man) database [2] and 220 non-disease-associated nsSNPs available in dbSNP database [1] which have no annotations in OMIM. All the nsSNPs in the test-set can be mapped to structures in the PDB (Dataset S1B and S1C) since *Bongo* uses structure as input.

For evaluation of *Bongo*, we calculated its sensitivity and specificity (with definitions explained in Table 2). By definition, if a method always classifies any mutation as ‘disease-associated’, it would achieve a sensitivity score of 100%. Similarly, a method could obtain a 100% specificity score by always predicting mutations as “non-disease-associated”. In order to avoid a biased analysis, we also calculated the PPV (positive predictive value) and NPV (negative predictive value; with definitions explained in Table 2); a better PPV or NPV implies a better performance in predicting positive or negative cases, respectively.

**Table 2:** Prediction of disease- and non-disease-associated nsSNPs

Methods	Sensitivity (%)	Specificity (%)	PPV (%)	NPV (%)
*Bongo*	28.1	82.4	78.5	34.5
*PolyPhen*	50.7	65.8	77.2	37.6
*PANTHER*	76.6	31.8	72.2	37.1

The test-set of disease-associated nsSNPs contains 506 nsSNPs that are covered in the OMIM database and can be mapped onto crystal structures in the PDB; the test-set of non-disease-associated nsSNPs contains 220 nsSNPs that are not covered in the OMIM database and can be mapped on crystal structures in the PDB. Sensitivity is defined as TP/(TP+FN); specificity is defined as TN/(TN+FP); PPV (positive predictive value) is defined as TP/(TP+FP); NPV (negative predictive value) is defined as TN/(TN+FN), where TP means the number of true positive predictions; FP means the number of false positive predictions; TN means the number of true negative predictions; FN means the number of false negative predictions.

The overall test results (Table 2) show that *Bongo* has PPV and NPV of 78.5% and 34.5%, respectively, compared to that of *PolyPhen* of 77.2% and 37.6%, respectively. This indicates that *Bongo* and *PolyPhen* have similar accuracy in predicting disease-associated nsSNPs. Given the fact that *PolyPhen* also exploits sequence information that may take account of protein interactions with various substrates, macromolecules and other ligands, we believe this shows the potential of using interaction networks which consider structure alone. The similar predictive values suggest that, although the mechanisms by which nsSNPs induce diseases are complicated, structural change is an important factor in most cases. This is consistent with a previous study that shows most deleterious nsSNPs affect protein stability but not functionality [14], which indicates that structural impact is a more important factor in causing disease. In order to assess the performance of *Bongo*, we also compared the use of *PANTHER* [36], which is verified to have higher accuracy than *PolyPhen* by using Hidden Markov Model (HMM) for sequence scoring. The result shows that *PANTHER* has the PPV and NPV values (There are 48 disease-associated and 22 non-disease-associated nsSNPs for which *PANTHER* did not find an HMM model to do prediction; those nsSNPs are excluded from the calculation of PPV and NPV values.) comparable to those of *PolyPhen* and *Bongo* (Table 2), which further verifies the evaluation.

In addition to the predictive value, *Bongo* has a low sensitivity (28.1%) compared to that of *PolyPhen* (50.7%) and *PANTHER* (76.6%), and its specificity (82.4%) is high compared to that of *PolyPhen* (65.8%) and *PANTHER* (31.8%). This suggests that, although *Bongo* has a similar predictive value to that of *PolyPhen* and *PANTHER*, *Bongo*'s high specificity and low sensitivity yields many less false positive predictions. We can thus be more confident about the cases that are predicted as disease-associated by *Bongo* than those predicted by *PolyPhen*. Regarding the low sensitivity of *Bongo*, we suppose this is due to the fact that *Bongo* is not able to predict mutations that only affect the function of proteins, e.g., the mutations in active or other interaction sites. We may improve *Bongo*'s ability in predicting functional site mutations in the future work.

Among the 506 disease-associated nsSNPs in our test-set, *Bongo* predicted 142 of them to cause structural effects, which suggests that about 28% of nsSNPs that are involved in Mendelian diseases resulting from single protein mutations may cause extensive structural effects in proteins. However, the figure for nsSNPs involved in multigenic diseases like diabetes may not be so high as they exist individually in the population as a whole at high levels, but contribute only rarely to multigenic diseases when occurring with several other nsSNPs.

### Conclusions

We have developed a method, *Bongo*, which uses graph theoretic measures to evaluate the structural impacts of single point mutations. Our approach has shown that identifying structurally important key residues in proteins is effective in predicting point mutations that cause extensive structural effects with a substantially lower false positive rate. Furthermore, our approach gives clues about the effects of nsSNPs on the structures of proteins, thus providing information complementary to methods based on sequence. By comparing our approach with *PolyPhen* and *PANTHER* in analyzing nsSNPs, we have also shown that structural information can provide results of quality comparable to those that use sequence and evolutionary information in predicting disease-associated nsSNPs.

## Methods

### Generating Residue–Residue Interaction Graphs

In the residue-residue interaction graphs, *Bongo* considers structural information including hydrogen bonds, π–π, π–cation, and hydrophobic interactions, as well as secondary structure information. (1) Hydrogen bond: we use *HBPLUS* [37] to calculate hydrogen bonds, using its default settings for positioning hydrogen and minimum angles formed by the donor and acceptor at the hydrogen. (2) π–π interaction: aromatic side chains are considered to have π–π interaction if they have less than 6 Å between any atoms. We note that more accurate criteria could be applied at the expense of the calculation speed with similar results. (3) π–cation interactions are identified on the condition that there is a cation within 7 Å of any side chain atoms of an aromatic ring such that the angle between the cation and the normal vector of the aromatic ring is within 60°. The criterion is only an approximate one in order to speed up the overall calculation without sacrificing the accuracy of calibration. (4) Hydrophobic interactions are weighted according to Voronoi surfaces between non-polar residues calculated by an in-house program, *Provat* [38], while hydrophobic cores are identified when a non-polar residue shares non-zero Voronoi surfaces with only non-polar residues. (5) Secondary structure elements are assigned by *DSSP* [39].

The weighting of the interactions were optimised by using the Least-Squares Optimisation Tool in MATLAB (http://www.mathworks.com/products/matlab/), where the best solution was chosen on the basis of the best calibration result over the eight mutations listed in Table 1. Although calibration was carried out against only eight mutations, the performance of *Bongo* on the 506 disease-associated nsSNPs, which are distributed in proteins from many different families, is comparable to that of *PolyPhen* (Table 2).

Since the mutant-type structures are not usually available, we generate them computationally using *Andante* [31]. Andante predicts the structure by using evolutionary information to define rotamers in clusters of side chains that are structurally compatible, so rearranging the local structure around the mutation site. It should be noted that *Bongo* does not benefit from sequence information by using *Andante*, since the rearrangement of local side chains modelled by *Andante* simply introduces a local rearrangement to the residue-residue interaction network of a protein, which does not affect the overall structure of the interaction graph and is independent of the process of selecting vertex cover.

All the structural information is transferred into graphs by using Graphviz (http://www.graphviz.org/), which is an open source graph visualization project from AT&T Research.

### The Graph Model of *Bongo*

*Bongo* derives the interaction graph of a protein by considering each residue as a vertex and each residue-residue interaction as an edge. More formally, an interaction graph *G* = (*V*,*E*) is a graph such that *V* is the set of residues and *E* is a set of edges. An edge (*u*,*v*) is defined between residue *u* and *v* if they exhibit one of the following interactions: backbone bonding, hydrogen bonds (H-bonds), π–π, π–cation, and hydrophobic interactions. Each edge is initially given a weight of 1. We then normalise interactions between two secondary structures by dividing the weight with the total number of cross-secondary structure interactions. Intra-secondary structure interactions are normalised in the same way. For interactions involving a group of residues, namely hydrophobic interactions, we normalise them by the Vonoroi surface area of each residues.

### Key Residues Provide an Approximation of the Vertex Cover with Minimal Weight

Since the key residues capture the vertices that are essential to maintain the interactions, we model them through the vertex cover set of the graph [29]. A vertex cover set *S* of a graph *G* = (*V*,*E*) is the set of vertices such that for every edge (*u*,*v*), either *u* or *v* is included in *S*. In the interaction graph terms, this amounts to picking a set of residues that covers every interaction in the graph. In *Bongo*, since the interactions are weighted, we consider the vertex cover problem *G* = (*V*,*E*,*c*) where *c*: *V* → *R* ^+^ is the function that assigns weight to each vertex. A vertex cover set is said to be *minimum* if it contains the set of vertices that covers all interactions with smallest possible weight.

### Selection Scheme That Identifies the Key Residues

**Figure 7:**
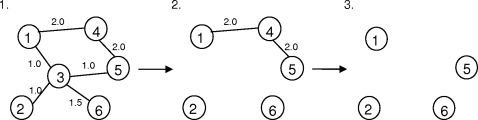
Scheme showing the algorithm that *Bongo* uses to identify key residues. In step 1, vertex 3 is identified as the first key residue since it has the greatest weight (4.5) of edges connected to it. In step 2, vertex 3 and all the edges connected to it are eliminated from the graph, and the next key residue is vertex 4 since it has the greatest weight (4) in the remaining graph. In step 3, there is no edge left in the graph thus the process of identifying key residues is terminated (if there are any edges left in the graph, the process of step 2 is repeated until no edge is left). Therefore, the key residues in this example are {3, 4}, and residue 3 is more important than residue 4 in terms of forming the graph.

The algorithm used to select key residues captures the concept of pulling out one piece each time in a tower of wooden pieces, with the difference that in our case the pieces pulled out are key pieces but not redundant ones (Figure 7):

1. Given a graph *G* = (*V*,*E*), pick the residue with highest weighting, if more than one residue has the same weighting, pick them all. That is, pick the set *U* = {*v*: *c*(*v*)≤*c*(*u*) ∀ *u*,*v* ∈ *V*}.
2. Remove all key residues and the edges connected to it. That is, replace the graph with *G* = (*W*, *F*) where *W* = *V*\*U* and *F* = *E*\{(*v*,*w*) ∈ *E*: *v* ∈ *U* ∨*w* ∈ *U*}.
3. Repeat (1) and (2) until no edge is left in the graph, i.e., *F* is empty.

The algorithm reflects the importance of key residues in order of selection: key residues selected in an earlier time are more important, in terms of having higher priorities in maintaining the interaction network, than others that are identified later. Since there is a specific order of choosing vertices, the approximate vertex cover chosen by *Bongo* for a specific graph will be the same when *Bongo* repeats the selection process again. Taking advantage of the priorities assigned to each key residue, *Bongo* eventually quantifies the effect of a point mutation by considering the priority of key residues affected.

## Supporting Information

Dataset S1 The 113 mutations that have negligible structural effects.
(0.02 MB PDF)
