# Supplementary material for: Prediction by Graph Theoretic Measures of Structural Effects in Proteins Arising from Non-Synonymous Single Nucleotide Polymorphisms
Source: PLoS Comput Biol. 2008 Jul 25;4(7):e1000135. doi: 10.1371/journal.pcbi.1000135 (PMC2447880; doi:10.1371/journal.pcbi.1000135)
Supplement: Dataset S1 — The 113 mutations that have negligible structural effects. (0.02 MB PDF) [file pcbi.1000135.s001.pdf]

Dataset S1A: The 113 mutations that have negligible structural effects.

Wild type (WT) protein	Mutant type (MT) protein	Residue number	WT amino acid	MT amino acid	WT Resolution (Å)	MT Resolution (Å)	RMSD (Å)	*Bongo* predict ion*
1bni	1ban	91	S	A	2.1	2.2	0.23	-
1bni	1bao	78	Y	F	2.1	2.2	0.34	-
1bni	1bns	26	T	A	2.1	2.05	0.23	-
1bni	1brg	7	F	L	2.1	2.2	0.37	Y
1bni	1brh	14	L	A	2.1	2.0	0.35	-
1bni	1bri	76	I	A	2.1	1.9	0.33	-
1bni	1brj	88	I	A	2.1	2.0	0.36	-
1bni	1brk	96	I	A	2.1	2.0	0.38	-
1bni	1bsa	51	I	V	2.1	2.0	0.33	-
1bni	1bsb	76	I	V	2.1	2.0	0.35	-
1bni	1bsc	88	I	V	2.1	2.0	0.35	-
1bni	1bse	89	L	V	2.1	2.0	0.34	-
1lz1	1gay	2	V	G	1.5	1.8	0.35	-
1lz1	1gaz	2	V	I	1.5	1.8	0.29	-
1lz1	1gb2	2	V	M	1.5	1.8	0.34	-
1lz1	1gb3	2	V	F	1.5	1.8	0.29	-
1lz1	1gf8	2	V	S	1.5	1.8	0.3	-
1lz1	1gf9	2	V	Y	1.5	1.8	0.29	-
1lz1	1gfa	2	V	D	1.5	1.8	0.28	-
1lz1	1gfe	2	V	N	1.5	1.8	0.29	-
1lz1	1gfg	2	V	R	1.5	1.8	0.31	-
1lz1	1gfh	74	V	Y	1.5	1.8	0.28	Y
1lz1	1gfj	74	V	D	1.5	1.8	0.3	-
1lz1	1gfk	74	V	N	1.5	1.8	0.26	-
1lz1	1gfu	110	V	D	1.5	1.8	0.32	-
1lz1	1gfv	110	V	N	1.5	1.8	0.27	-
1lz1	1lhh	110	V	P	1.5	1.8	0.28	-
1lz1	1lhi	71	P	G	1.5	1.8	0.24	-
1lz1	1lhj	103	P	G	1.5	1.8	0.23	-
1lz1	1lhk	91	D	P	1.5	1.8	0.27	-
1lz1	1lhl	47	A	P	1.5	1.8	0.27	-
1lz1	1oua	56	I	T	1.5	1.8	0.25	-
1lz1	1oub	100	V	A	1.5	1.8	0.26	-
1lz1	1oug	2	V	A	1.5	1.8	0.23	-
1lz1	1ouh	74	V	A	1.5	1.8	0.27	-
1lz1	1oui	93	V	A	1.5	1.8	0.26	-
1lz1	1ouj	99	V	A	1.5	1.8	0.28	-
1lz1	1tcy	63	Y	F	1.5	1.7	0.12	Y
1lz1	1wqm	124	Y	F	1.5	1.8	0.24	-
1lz1	1wqn	20	Y	F	1.5	1.8	0.25	-
1lz1	1wqo	38	Y	F	1.5	1.8	0.27	-
1lz1	1wqp	45	Y	F	1.5	1.8	0.24	-

1lz1	1yam	106	I	V	1.5	1.8	0.26	-
1lz1	1yan	23	I	V	1.5	1.8	0.27	-
1lz1	1yao	56	I	V	1.5	1.8	0.24	-
1lz1	1yap	59	I	V	1.5	1.8	0.26	-
1lz1	1yaq	89	I	V	1.5	1.8	0.25	-
1lz1	2hea	106	I	A	1.5	1.8	0.28	-
1lz1	2heb	23	I	A	1.5	1.8	0.3	-
1lz1	2hec	56	I	A	1.5	1.8	0.23	-
1lz1	2hed	59	I	A	1.5	1.8	0.27	-
1lz1	2hee	59	I	G	1.5	1.8	0.25	-
1lz1	2hef	89	I	A	1.5	1.8	0.23	-
1vqb	1vqg	47	I	L	1.8	1.8	0.07	-
1vqb	1vqh	47	I	M	1.8	1.8	0.08	-
1vqb	1vqi	47	I	V	1.8	1.8	0.09	-
1vqb	1vqj	35	V	I	1.8	1.8	0.07	-
1vqb	1yhb	41	Y	F	1.8	2.2	0.25	-
2ci2	1coa	76	I	V	2.0	2.2	0.18	-
2lzm	1dya	131	V	D	1.7	1.9	0.15	-
2lzm	1dyb	131	V	G	1.7	1.75	0.15	-
2lzm	1dyc	131	V	I	1.7	2.1	0.15	-
2lzm	1dyd	131	V	L	1.7	2.1	0.17	-
2lzm	1dye	131	V	S	1.7	1.8	0.14	-
2lzm	1dyf	131	V	M	1.7	1.9	0.15	-
2lzm	1dyg	131	V	E	1.7	2.1	0.18	-
2lzm	1l00	105	Q	A	1.7	1.9	0.26	-
2lzm	1l02	157	T	A	1.7	1.7	0.09	-
2lzm	1l03	157	T	C	1.7	1.7	0.13	-
2lzm	1l04	157	T	D	1.7	1.7	0.1	-
2lzm	1l06	157	T	E	1.7	1.7	0.1	-
2lzm	1l12	157	T	N	1.7	1.7	0.07	-
2lzm	1l13	157	T	R	1.7	1.7	0.07	-
2lzm	1l14	157	T	S	1.7	1.7	0.07	-
2lzm	1l15	157	T	V	1.7	1.7	0.07	-
2lzm	1l16	156	G	D	1.7	1.7	0.14	-
2lzm	1l17	3	I	V	1.7	1.7	0.14	-
2lzm	1l18	3	I	Y	1.7	1.7	0.25	-
2lzm	1l19	38	S	D	1.7	1.7	0.13	-
2lzm	1l20	144	N	D	1.7	1.85	0.15	-
2lzm	1l21	144	N	D	1.7	1.85	0.15	-
2lzm	1l22	124	K	G	1.7	1.7	0.19	-
2lzm	1l23	77	G	A	1.7	1.7	0.14	-
2lzm	1l24	82	A	P	1.7	1.7	0.13	-
2lzm	1l33	131	V	A	1.7	1.7	0.15	-
2lzm	1l34	96	R	H	1.7	1.9	0.17	-
2lzm	1l37	115	T	E	1.7	1.85	0.15	-
2lzm	1l38	123	Q	E	1.7	1.8	0.13	-
2lzm	1l42	16	K	E	1.7	1.8	0.15	-

2lzm	1l44	119	R	E	1.7	1.7	0.16	-
2lzm	1l45	135	K	E	1.7	1.7	0.15	-
2lzm	1l46	147	K	E	1.7	1.7	0.16	-
2lzm	1l47	154	R	E	1.7	1.7	0.16	-
2lzm	1l48	98	A	V	1.7	1.7	0.2	-
2lzm	1l52	152	T	S	1.7	1.7	0.14	-
2lzm	1l53	149	V	C	1.7	1.85	0.31	-
2lzm	1l56	60	K	P	1.7	1.8	0.18	-
2lzm	1l57	116	N	D	1.7	1.9	0.17	-
2lzm	1l60	113	G	A	1.7	1.7	0.21	-
2lzm	1l69	133	L	A	1.7	1.9	0.17	-
2lzm	1l96	3	I	P	1.7	2.0	0.21	-
2lzm	1l98	105	Q	E	1.7	1.8	0.26	-
2lzm	1l99	105	Q	G	1.7	1.95	0.3	-
2rn2	1gob	77	G	A	1.48	2.0	0.28	-
2rn2	1kva	134	D	A	1.48	1.8	0.26	-
2rn2	1lav	74	V	L	1.48	1.8	0.16	-
2rn2	1law	74	V	I	1.48	1.8	0.17	-
2rn2	1rbr	62	H	P	1.48	1.8	0.2	-
2rn2	1rbt	95	K	G	1.48	1.8	0.16	-
2rn2	1rbu	95	K	N	1.48	1.8	0.17	-
2rn2	1rbv	95	K	A	1.48	1.8	0.13	-
2rn2	1rda	10	D	N	1.48	2.15	0.23	-
2rn2	1rdb	48	E	Q	1.48	1.9	0.21	-

* Mutations that are predicted to cause structural effects are indicated as ‘Y‘, whereas those predicted to cause no structural effects are indicated as ‘-‘.

Dataset S1B: Disease-associated nsSNPs that are predicted in this paper. The information of the nsSNPs is shown in the following as *PDB ID_chain-name_nsSNP*:

1A00_C_A110D	1A00_C_A120E	1A00_C_A12D	1A00_C_A130D	1A00_C_A130P	1A00_C_A21D	1A00_C_A21P
1A00_C_A26E	1A00_C_A63D	1A00_C_A88S	1A00_C_D126V	1A00_C_D126Y	1A00_C_D6G	1A00_C_D6N
1A00_C_D6V	1A00_C_D6Y	1A00_C_D94Y	1A00_C_E116A	1A00_C_E27D	1A00_C_E27G	1A00_C_E27V
1A00_C_E30K	1A00_C_F43L	1A00_C_G18D	1A00_C_G18R	1A00_C_G51D	1A00_C_H103R	1A00_C_H112D
1A00_C_H45Q	1A00_C_H45R	1A00_C_H50R	1A00_C_H58Y	1A00_C_H87N	1A00_C_H87R	1A00_C_K11E
1A00_C_K127N	1A00_C_K139E	1A00_C_K139T	1A00_C_K56R	1A00_C_K56T	1A00_C_K61N	1A00_C_K61T
1A00_C_K99E	1A00_C_L109R	1A00_C_L113H	1A00_C_L125P	1A00_C_L129P	1A00_C_L136M	1A00_C_L136P
1A00_C_L2R	1A00_C_L34R	1A00_C_L48R	1A00_C_L80R	1A00_C_L91P	1A00_C_M76K	1A00_C_M76T
1A00_C_N97K	1A00_C_P114L	1A00_C_P114R	1A00_C_P37R	1A00_C_P77R	1A00_C_P95A	1A00_C_P95T
1A00_C_R141C	1A00_C_R141H	1A00_C_R141L	1A00_C_R141P	1A00_C_R92Q	1A00_C_S102R	1A00_C_S131P
1A00_C_S133R	1A00_C_S138P	1A00_C_S49R	1A00_C_S81C	1A00_C_S84R	1A00_C_T41S	1A00_C_V121M
1A00_C_V135E	1A00_C_V62M	1A00_C_W14R	1A00_C_Y140H	1A00_C_Y24H	1A00_D_D_A10D	1A00_D_D_A128D
1A00_D_D_A129P	1A00_D_D_A129V	1A00_D_D_A135E	1A00_D_D_A135P	1A00_D_D_A138P	1A00_D_D_A140D	1A00_D_D_A140T
1A00_D_D_A140V	1A00_D_D_A142D	1A00_D_D_A27D	1A00_D_D_A27S	1A00_D_D_A27V	1A00_D_D_A62D	1A00_D_D_A62P
1A00_D_D_A70D	1A00_D_D_A86D	1A00_D_D_C112R	1A00_D_D_C112Y	1A00_D_D_C93R	1A00_D_D_D21G	1A00_D_D_D21H
1A00_D_D_D21N	1A00_D_D_D21Y	1A00_D_D_D52A	1A00_D_D_D52H	1A00_D_D_D94G	1A00_D_D_D94H	1A00_D_D_D94N
1A00_D_D_D99E	1A00_D_D_E101D	1A00_D_D_E101G	1A00_D_D_E101K	1A00_D_D_E101Q	1A00_D_D_E22A	1A00_D_D_E22G
1A00_D_D_E22K	1A00_D_D_E22Q	1A00_D_D_E22V	1A00_D_D_E26K	1A00_D_D_E26V	1A00_D_D_E6A	1A00_D_D_E6K
1A00_D_D_E6Q	1A00_D_D_E6V	1A00_D_D_E7G	1A00_D_D_E7K	1A00_D_D_E90D	1A00_D_D_E90K	1A00_D_D_F103L
1A00_D_D_F41Y	1A00_D_D_F42L	1A00_D_D_F45S	1A00_D_D_F71S	1A00_D_D_G107R	1A00_D_D_G119A	1A00_D_D_G136D
1A00_D_D_G24D	1A00_D_D_G24R	1A00_D_D_G24V	1A00_D_D_G25D	1A00_D_D_G25R	1A00_D_D_H116Q	1A00_D_D_H143D
1A00_D_D_H143P	1A00_D_D_H143Q	1A00_D_D_H143R	1A00_D_D_H146D	1A00_D_D_H146L	1A00_D_D_H146P	1A00_D_D_H146Q
1A00_D_D_H2L	1A00_D_D_H2Q	1A00_D_D_H2R	1A00_D_D_H2Y	1A00_D_D_H63Y	1A00_D_D_H92D	1A00_D_D_H92N
1A00_D_D_H92P	1A00_D_D_H92Q	1A00_D_D_H97L	1A00_D_D_H97P	1A00_D_D_H97Q	1A00_D_D_H97Y	1A00_D_D_K120E
1A00_D_D_K120I	1A00_D_D_K120Q	1A00_D_D_K132N	1A00_D_D_K132Q	1A00_D_D_K144E	1A00_D_D_K59E	1A00_D_D_K61E
1A00_D_D_K61M	1A00_D_D_K61N	1A00_D_D_K66T	1A00_D_D_K82M	1A00_D_D_K95M	1A00_D_D_K95N	1A00_D_D_L110P
1A00_D_D_L114M	1A00_D_D_L114P	1A00_D_D_L141R	1A00_D_D_L28P	1A00_D_D_L31P	1A00_D_D_L48P	1A00_D_D_L68H
1A00_D_D_L68P	1A00_D_D_L75P	1A00_D_D_L78R	1A00_D_D_L81R	1A00_D_D_L88P	1A00_D_D_L88R	1A00_D_D_L91P
1A00_D_D_L91R	1A00_D_D_L96V	1A00_D_D_N102S	1A00_D_D_N102Y	1A00_D_D_N108K	1A00_D_D_N139D	1A00_D_D_N139K
1A00_D_D_N139Y	1A00_D_D_P100L	1A00_D_D_P100R	1A00_D_D_P36R	1A00_D_D_P36S	1A00_D_D_P36T	1A00_D_D_P51R
1A00_D_D_Q131E	1A00_D_D_Q131K	1A00_D_D_Q131P	1A00_D_D_Q131R	1A00_D_D_Q39E	1A00_D_D_Q39R	1A00_D_D_R104T
1A00_D_D_R30S	1A00_D_D_S49F	1A00_D_D_S89N	1A00_D_D_S89R	1A00_D_D_T38N	1A00_D_D_V109M	1A00_D_D_V111A
1A00_D_D_V11D	1A00_D_D_V11I	1A00_D_D_V126A	1A00_D_D_V126E	1A00_D_D_V126G	1A00_D_D_V134E	1A00_D_D_V34F
1A00_D_D_V60A	1A00_D_D_V67A	1A00_D_D_V67M	1A00_D_D_V98G	1A00_D_D_Y130D	1A00_D_D_Y130S	1A00_D_D_Y145C
1A00_D_D_Y145H	1A00_D_D_Y35F	1A01_C_A5D	1A01_C_A5P	1A01_C_D64Y	1A01_C_D75A	1A01_C_D75H
1A01_C_H72R	1A01_C_K40M	1A01_C_N78H	1A01_C_N78K	1A01_C_R31S	1A01_D_D_G29D	1A01_D_D_H117P
1A01_D_D_H117R	1A01_D_D_K17E	1A01_D_D_K17N	1A01_D_D_K17Q	1A01_D_D_K8Q	1A01_D_D_K8T	1A01_D_D_N19D
1A01_D_D_N19K	1A01_D_D_N19S	1A01_D_D_P124Q	1A01_D_D_P124R	1A01_D_D_P124S	1A01_D_D_T123I	1A01_D_D_V18M
1A01_D_D_V23D	1A01_D_D_V23F	1A01_D_D_V23G	1A01_D_D_V54D	1A0U_C_A71E	1A0U_C_A71V	1A0U_C_D74A
1A0U_C_D74G	1A0U_C_D74N	1A0U_C_E23G	1A0U_C_E23K	1A0U_C_H20Q	1A0U_C_H20R	1A0U_C_K16M
1A0U_C_K16N	1A0U_C_K90M	1A0U_C_P44L	1A0U_C_P44R	1A0U_D_D_D79Y	1A0U_D_D_G74R	1A0U_D_D_G74V
1A3N_D_V133L	1A3O_D_E43Q	1A8E_-_G258S	1ABY_A_G57R	1ABY_A_Q54R	1ABY_D_D_K65M	1ABY_D_D_K65N
1ABY_D_D_K65Q	1ABY_D_D_L14P	1ABY_D_D_L14R	1ABY_D_D_T84I	1AJ9_B_D73G	1AJ9_B_D73V	1AJ9_B_D73Y
1AJ9_B_N57K	1AJ9_B_V20M	1ALD_-_D128G	1APY_C_A78V	1APY_C_G37D	1APY_C_S49P	1APY_D_D_G279R
1AZV_B_A4T	1AZV_B_A4V	1AZV_B_C6F	1AZV_B_D90A	1AZV_B_E100G	1AZV_B_E21K	1AZV_B_G16S
1AZV_B_G72S	1AZV_B_G85R	1AZV_B_H46R	1AZV_B_I113T	1AZV_B_I151T	1AZV_B_L106V	1AZV_B_L144F
1AZV_B_L144S	1BHG_A_A354V	1BHG_A_A619V	1BHG_A_R382C	1BHG_A_R611W	1BM7_A_A91S	1BM7_A_E89K
1BM7_A_K70N	1BM7_A_S77Y	1BZ4_A_G127D	1BZ4_A_R134Q	1BZ8_B_F64L	1BZ8_B_I84S	1BZ8_B_P102R
1BZ8_B_V30A	1BZ8_B_V30L	1BZ8_B_V30M	1BZE_B_S50I	1BZE_B_S50R	1BZY_D_D176V	1BZY_D_D176Y
1BZY_D_D193E	1BZY_D_D193N	1BZY_D_D51G	1BZY_D_H203D	1BZY_D_H203R	1BZY_D_M56T	1BZY_D_R44K
1BZY_D_S161R	1BZY_D_V52A	1BZY_D_V52M	1C9Y_A_E87K	1C9Y_A_G162R	1C9Y_A_K46R	1C9Y_A_L111P
1C9Y_A_L148F	1C9Y_A_L45P	1C9Y_A_L45V	1C9Y_A_M206R	1C9Y_A_N47I	1C9Y_A_P225L	1C9Y_A_P225T
1C9Y_A_Q216E	1C9Y_A_R129H	1C9Y_A_R277Q	1C9Y_A_R277W	1C9Y_A_R40C	1C9Y_A_R40H	1CBL_D_D47A
1CBL_D_D47G	1CBL_D_D47Y	1CBL_D_S44C	1CBM_D_A115D	1CBM_D_A115P	1DEH_B_R369C	1DEH_B_R47H
1EFV_A_G116R	1EFV_A_T266M	1EFV_A_V157G	1EFV_B_B_R164Q	1EGC_D_C219R	1EGC_D_I350T	1EGC_D_M124I
1EGD_D_G242R	1FQY_A_A45V	1GBN_C_A270P	1GBN_C_H319Y	1GBN_C_N54K	1GBN_C_P241L	1GBN_C_R154L

1GBN_C_R180T	1GBN_C_Y55H	1GBV_D_G46E	1GLI_D_A13D	1GLI_D_E121A	1GLI_D_E121G	1GLI_D_E121K
1GLI_D_E121Q	1GLI_D_E121V	1GUX_B_C712R	1GUX_B_R661W	1HBS_G_A82D	1HBS_G_D47A	1HBS_G_D47G
1HBS_G_D47Y	1HBS_G_G59D	1HBS_G_G59V	1HBS_H_H_G16D	1HBS_H_H_G16R	1HBS_H_H_P5R	1HBS_H_H_S9C
1HCO_B_P58R	1HRY_A_F54S	1HRY_A_G40R	1HRY_A_I13T	1HRY_A_I35M	1HRY_A_K51I	1HRY_A_M9I
1HRY_A_V5A	1HRY_A_V5L	1HTI_B_C41Y	1HTI_B_E104D	1HTI_B_F240L	1HTI_B_G122R	1HTI_B_I170V
1LE2_-_Q81K	1OAT_C_A226V	1OAT_C_C394R	1OAT_C_C93F	1OAT_C_G353D	1OAT_C_G375A	1OAT_C_L402P
1OAT_C_P417L	1OAT_C_R250P	1OAT_C_V332M	1OLG_C_G325V	1PAH_-_R158Q	1QAB_A_Y69H	1SOS_J_G41D
1SOS_J_G41S	1SOS_J_G93A	1SOS_J_G93C	1SOS_J_G93D	1SOS_J_G93R	1SOS_J_G93V	1SOS_J_S134N
1SPD_B_A145T	1TSR_A_A138P	1TSR_A_C242F	1TSR_A_C242S	1TSR_A_E258D	1TSR_A_E258K	1TSR_A_G245A
1TSR_A_G245C	1TSR_A_G245D	1TSR_A_G245S	1TSR_A_G245V	1TSR_A_H168R	1TSR_A_I195T	1TSR_A_L252P
1TSR_A_L257P	1TSR_A_M133T	1TSR_A_R175C	1TSR_A_R175G	1TSR_A_R175H	1TSR_A_R175L	1TSR_A_R175P
1TSR_A_R181L	1TSR_A_R248A	1TSR_A_R248G	1TSR_A_R248L	1TSR_A_R248Q	1TSR_A_R248W	1TSR_A_R249G
1TSR_A_R249S	1TSR_A_R273A	1TSR_A_R273C	1TSR_A_R273G	1TSR_A_R273H	1TSR_A_R280I	1TSR_A_R280K
1TSR_A_R280T	1TSR_A_R282W	1TSR_A_S241F	1TSR_A_V143A	1TSR_A_V157D	1TSR_A_V157S	1TSR_A_V272L
1URO_A_E167K	1URO_A_G281E	1URO_A_L195F	1URO_A_M165R	1URO_A_P62L	1URO_A_R292G	1URO_A_R332H
1YCS_A_P151A	1YCS_A_P151S	1YCS_A_P151T	2CAB_-_G253R	2OAT_C_L437F	2OAT_C_R271K	6PAX_A_G61V
6PAX_A_R23G	6PAX_A_V123D					

Dataset S1C: Non-disease-associated nsSNPs that are predicted in this paper. The information of the nsSNPs is shown in the following as *PDB ID_chain-name_nsSNP*:

13GS_A_A113V	1A02_N_H419Y	1A0L_D_D143N	1A0L_D_G37D	1A0L_D_R187Q	1A0L_D_T122A	1A0L_D_T186S
1A49_A_R338P	1A6D_B_I101V	1AD3_A_R11P	1ADL_-_T103P	1AG8_D_A69V	1AG8_D_R90L	1AII_-_I220N
1AII_-_P252L	1AK6_-_R95S	1ALD_-_F144I	1ANN_-_R43C	1AV1_D_A194G	1AV1_D_D102H	1AV1_D_K107M
1AV1_D_R160P	1AX8_-_V89M	1AXC_E_S39R	1BD9_B_H145Y	1BD9_B_H26N	1BD9_B_V34A	1BGY_B_R169Q
1BI9_D_S107G	1BMO_B_R151G	1BOY_-_R131W	1BQQ_M_D273N	1BRU_P_N233S	1BSX_B_T337I	1BUC_B_G343D
1BY7_A_I114T	1C46_A_T70N	1C5G_A_H25P	1C5G_A_R209H	1C5G_A_T255N	1C9Y_A_K46R	1C9Y_A_L111P
1CB5_B_I443V	1CJM_A_E151D	1CJM_A_E151Q	1CJM_A_G170R	1CJM_A_H149Y	1CJY_A_M130I	1CJY_A_R657K
1CP3_B_G66R	1CTS_-_P15L	1CTS_-_S378R	1CW3_A_E487K	1CX2_D_E486G	1CX2_D_V509A	1D1S_D_G79A
1D2V_D_N434Y	1DE4_G_H41D	1DE4_G_M13T	1DG9_A_Q105R	1DO8_C_G450E	1DQV_A_P343T	1DQV_A_V316L
1DUJ_A_L74M	1DVA_I_R204Q	1DVA_I_V95D	1DXT_D_E7V	1E03_L_T115A	1E1Q_B_G130S	1E1Q_B_G92R
1E1Q_B_V88M	1E1Y_A_N187K	1E4K_C_I85V	1E4K_C_S44N	1E9L_A_G102S	1E9N_B_I64V	1E9N_B_Q51H
1ED3_D_H3R	1EEM_A_A140D	1EJ1_B_S53T	1EKG_A_S202C	1EL3_A_G203S	1EL3_A_H41L	1EL3_A_I14F
1EL3_A_L72V	1EL3_A_T287I	1EM6_A_R714S	1EM6_A_V230E	1ESL_-_S128R	1EZF_A_K45R	1F2Q_A_K59R
1F2Q_A_S76N	1F45_B_M191T	1F4R_A_R120C	1F4R_A_R141Q	1F5F_A_P156L	1F5N_A_I78V	1F5N_A_K429R
1F5N_A_N513Y	1F5N_A_T203A	1F5N_A_T481I	1F6W_A_A436G	1F6W_A_A436T	1F6W_A_D438E	1F6W_A_K445E
1F8U_A_D74G	1F8U_A_H322N	1F8U_A_V302E	1FBL_-_D252G	1FJM_B_V250L	1FP3_B_D274G	1FPU_A_L429V
1FR8_B_H261R	1FUJ_C_A119T	1FUJ_C_T120S	1FUU_B_P294L	1G0U_L_G170V	1G0U_Y_L146M	1G0W_A_L360F
1G0X_A_D123E	1G0X_A_D129E	1G6V_A_N253D	1G8M_B_T117S	1GGL_B_D18N	1GGL_B_M114L	1GUH_B_A12T
1GUH_B_A216S	1GUH_B_C112S	1GUH_B_E210A	1GUH_B_F10S	1GUH_B_I128L	1GUH_B_I128T	1GUH_B_K117Q
1GUH_B_K125Q	1GUH_B_P113Q	1GUH_B_T19I	1GUH_B_V111L	1GUH_B_V149A	1H4U_A_V408G	1H7X_C_R886H
1H7X_C_V995F	1HCI_A_Q516R	1HDM_B_I172T	1HDO_A_A70T	1HLC_B_V119I	1HML_-_I27V	1HQ3_A_N110K
1HSZ_B_N56K	1HT0_B_Q271R	1HTI_B_G120A	1HTI_B_G9V	1HW8_B_I638V	1HWG_A_R64W	1HYN_Q_K56E
1HYN_Q_R112S	1HYR_C_D113H	1HYR_C_L122V	1I1I_P_S394G	1I5I_A_F5L	1I9S_A_M30I	1IAR_B_I50V
1IB1_D_V142E	1IJQ_A_I567L	1IJQ_A_P664S	1IJQ_A_S609T	1IJQ_A_V409M	1IJQ_A_V447I	1IJQ_A_W422C
1IJQ_B_S566G	1IM9_E_E253Q	1IRL_-_L18R	1JEN_A_R128Q	1JEN_A_R128W	1KLT_-_H57R	1LFO_-_T94A
1LJR_B_A21T	1LJR_B_D140N	1LJR_B_E172K	1LJR_B_M139I	1LJR_B_V118M	1LVK_-_N219S	1MHL_D_I551V
1MKP_-_G296S	1NDH_-_T88S	1PKM_-_C48R	1POE_B_L11F	1QLP_A_R101H	1QLR_C_A9G	1QLR_C_D50G
1QLR_C_G24R	1QLR_C_P95H	1QLR_C_S67P	1RP1_-_S12F	1RRG_B_S147F	1SGF_Z_E128Q	1SGF_Z_E23K
1SGF_Z_V176E	1SLM_-_R48K	1TND_A_G179D	1TND_A_S224T	1TRN_B_Y172C	1WAB_-_R214G	1XBR_A_G175D
1XNA_A_R7L	1XNA_A_V10M	1ZXQ_-_R175H	2ADA_-_K171N	2BN2_A_P51L	2GTU_B_A129E	2GTU_B_M133K
2MYS_A_G699R	2SHP_A_L88V	2SHP_A_V148E	2SRC_-_A434D	2SRC_-_D235V	2SRC_-_I441F	3ADK_-_I146M
3ADK_-_R97Q	3GTU_D_V223I	4P2P_-_N67K				
